# Development and Validation of a Nomogram Incorporating Colloid Osmotic Pressure for Predicting Mortality in Critically Ill Neurological Patients

**DOI:** 10.3389/fmed.2021.765818

**Published:** 2021-12-24

**Authors:** Bo Lv, Linhui Hu, Heng Fang, Dayong Sun, Yating Hou, Jia Deng, Huidan Zhang, Jing Xu, Linling He, Yufan Liang, Chunbo Chen

**Affiliations:** ^1^Department of Critical Care Medicine, Guangdong Provincial People's Hospital, Guangdong Academy of Medical Sciences, Guangzhou, China; ^2^The Second School of Clinical Medicine, Southern Medical University, Guangzhou, China; ^3^Department of General Practice, Guangdong Provincial Geriatrics Institute, Guangdong Provincial People's Hospital, Guangdong Academy of Medical Sciences, Guangzhou, China; ^4^Department of Critical Care Medicine, Maoming People's Hospital, Maoming, China; ^5^Department of Clinical Research Center, Maoming People's Hospital, Maoming, China; ^6^Department of Emergency, Longgang District Central Hospital, Shenzhen, China; ^7^Department of Oncology, Maoming People's Hospital, Maoming, China; ^8^Department of Intensive Care Unit of Cardiac Surgery, Guangdong Cardiovascular Institute, Guangdong Provincial People's Hospital, Guangdong Academy of Medical Sciences, Guangzhou, China

**Keywords:** colloid osmotic pressure, mortality, critically ill neurological patients, nomogram, predicting

## Abstract

**Backgrounds:** The plasma colloid osmotic pressure (COP) values for predicting mortality are not well-estimated. A user-friendly nomogram could predict mortality by incorporating clinical factors and scoring systems to facilitate physicians modify decision-making when caring for patients with serious neurological conditions.

**Methods:** Patients were prospectively recruited from March 2017 to September 2018 from a tertiary hospital to establish the development cohort for the internal test of the nomogram, while patients recruited from October 2018 to June 2019 from another tertiary hospital prospectively constituted the validation cohort for the external validation of the nomogram. A multivariate logistic regression analysis was performed in the development cohort using a backward stepwise method to determine the best-fit model for the nomogram. The nomogram was subsequently validated in an independent external validation cohort for discrimination and calibration. A decision-curve analysis was also performed to evaluate the net benefit of the insertion decision using the nomogram.

**Results:** A total of 280 patients were enrolled in the development cohort, of whom 42 (15.0%) died, whereas 237 patients were enrolled in the validation cohort, of which 43 (18.1%) died. COP, neurological pathogenesis and Acute Physiology and Chronic Health Evaluation II (APACHE II) score were predictors in the prediction nomogram. The derived cohort demonstrated good discriminative ability, and the area under the receiver operating characteristic curve (AUC) was 0.895 [95% confidence interval (CI), 0.840–0.951], showing good correction ability. The application of this nomogram to the validation cohort also provided good discrimination, with an AUC of 0.934 (95% CI, 0.892–0.976) and good calibration. The decision-curve analysis of this nomogram showed a better net benefit.

**Conclusions :** A prediction nomogram incorporating COP, neurological pathogenesis and APACHE II score could be convenient in predicting mortality for critically ill neurological patients.

## Introduction

Cerebral oedema in patients with critical neurological status may be caused by multiple pathological mechanisms associated with primary and secondary injury patterns. As the internal pressure in the skull increases, intracranial hypertension can cause brain tissues to shift, leading to disability or death (1–4). Owing to the importance of intracranial pressure (ICP) monitoring in the care of patients with critical neurological status and its relationship with overall prognosis, ICP monitoring is included in many guidelines for treating brain injury (5–8). Nevertheless, ICP monitoring is limited by the invasive nature of specific techniques and the high risk–benefit ratios used in certain patient populations (9). Although ICP monitoring is often used in developed countries, it is still grossly inadequate in developing areas. Consequently, it is meaningful to explore the use of existing biomarkers to detect the prognosis and overall situation of patients with craniocerebral injury.

A study revealed that serum albumin (sALB) and Glasgow coma scale (GCS) scores were predictors of survival in patients with neurological condition requiring intensive care (10). Additionally, sALB is the main component of maintaining plasma colloid osmotic pressure (COP). The maintenance of COP is vital for controlling the distribution of extracellular fluid between blood vessels and extravascular compartments (11–14). Thus, theoretically, COP might be used to assess the overall pathophysiological status of patients with critical neurological conditions in some cases. Furthermore, with the advantages of short intervals at the bedside, COP would be a helpful, convenient and safe tool to enable regular monitoring, especially in resource-constrained areas without advanced and expensive equipment. These features have established that COP can predict the prognosis and identify related prognostic characteristics.

However, predictive models for critically ill neurological patients are non-existent, even though several predictive models have been constructed for clinical subgroups, such as for patients with traumatic brain injury and subarachnoid hemorrhage (15, 16). A study revealed that combining biomarkers and clinical factors into a model could be accurate and convenient in predicting prognoses (17, 18). A nomogram for predicting mortality would greatly help the physician to make decisions and would be essential for risk stratification in quality improvement and clinical research. With this background, this study aimed to establish a nomogram for predicting mortality in critically ill neurological patients by incorporating clinical factors and scoring systems.

## Materials and Methods

### Setting, Participants, and Data Collection

This two-stage prospective, observational study was performed in two tertiary hospitals in Guangdong Province, China. In the first stage, all patients aged >18 years were recruited from the neurointensive care unit (NICU) in Guangdong Provincial People's Hospital between March 2017 and September 2018 as the development cohort. Similarly, in the second stage, patients with neurological diseases who were aged over 18 years and admitted in the emergency intensive care unit (EICU) or the emergency department of Longgang District Central Hospital were prospectively collected from October 2018 to June 2019 as the validation cohort. Patients with incomplete data were excluded from the analysis. The outcome was mortality in the NICU, EICU or emergency department. All patients were recruited after signing informed consent. This study was approved by the Ethics Committee of the participating centers and complied with the criteria of the Helsinki Declaration.

### Sample Size Consideration

The rule of thumb recommended by Peduzzi et al. was used to calculate the sample size (19), that is, events per variable being 10 or more significant in the setting of the multivariate regression model. Thus, ~3–4 variables might be obtained in developing a model. Consequently, a sample size of 30 (3^*^10) to 40 (4^*^10) participants who died was required to predict the outcome. Finally, a minimum sample of 40 positive events, that is, patients with neurological diseases who died in the NICU, EICU or emergency department, was adopted to conduct this study.

### Sample Measurements and Data Collection

All blood samples were gathered within 1 h after admission to the NICU, EICU or emergency department. Serum total protein (sTP), sALB, serum hemoglobin, fibrinogen, crystal osmotic pressure, serum sodium, blood glucose, serum potassium, serum creatinine, blood urea nitrogen and blood calcium were measured at the central laboratory of the hospitals using a standard protocol within 24 h after collection.

The COP detector (ONKOMETER BMT923, BMT MESSTECHNIK GMBH, German) was used to measure the COP. First, 0.5 ml of blood collected from the radial vein was added to the detector, the blood was waited to settle and evenly distribute for 15 s. Then, an additional 0.2 ml of venous blood was added to enable measurement of the COP (20, 21). The COP was measured from samples obtained within 1 h after admission.

Patients were classified into different neurological pathogenesis according to pathophysiological mechanisms reflected by the diagnosis at the time of admission. Moreover, data concerning demographic characteristics (age and gender), pre-existing comorbidities (hypertension and diabetes mellitus), neurological pathogenesis (vascular, tumorous, infectious, traumatic, metabolic and toxic), Acute Physiology and Chronic Health Evaluation II (APACHE II score) and GCS score were documented on admission. In addition, after discharge from the NICU, EICU or emergency department, prognosis information, including the duration of mechanical ventilation, length of stay and mortality, was recorded.

### Statistical Analysis

Shapiro–Wilk test was employed to identify the normal distribution of continuous variables, expressed as the median and standard deviation. Wilcoxon–Mann–Whitney *U*-test was performed on the skew distribution, defined as the median and interquartile range. Descriptive statistics for categorical variables were described in the form of frequency (percentage) and were compared by the Pearson χ2 test or Fisher's exact test, appropriately. Possible confounding factors and clinically significant factors were included in the univariate analysis. Variables with *P*-values < 0.05 in the univariate analysis were considered in the multivariate model. Spearman correlation and Belsley collinearity test were used to evaluate the collinearity among all covariates. Multivariate logistic regression analysis used by a backward stepwise approach was first conducted to determine the reduced model in the development cohort to establish a predictive nomogram of mortality. In addition, to visualize the linear relationship between COP and predicted ICU mortality, a scatter plot with a smooth curve derived by a method of generalized additive model was depicted. Covariates were COP, neurological pathogenesis, sTP, sALB and APACHE II score. Estimated odds ratios (OR) as well as 95% confidence intervals (CI) were calculated. Discrimination was assessed using the area under the receiver operating characteristic curve (AUC) resulting from the conventional receiver operating characteristic (ROC) curves. The non-parametric approach of DeLong and Clarke-Pearson was utilized to contradistinguish the AUC values in those two models. The development and validation models were calculated for accuracy, sensitivity, specificity, positive predictive value (PPV) and negative predictive value (NPV). Finally, the nomogram containing the identified predictive variables was generated from the reduced model. Primarily, a vertical line was drawn from the factor to the point axis to calculate the points of each predictor in the nomogram. Subsequently, all points from all predictors were utilized to identify the total points. Thus, the estimated probability of mortality could be gained using a vertical line drawn from the whole point axis to the risk of mortality axis.

Bootstrapping methods were used to perform the validation and calibration of the best-fit model and nomogram. Approximately 1,000 patients were used in the bootstrap method to report the bootstrap-corrected AUC and 95% CI. The calibration plots of the nomogram were assessed by using the Hosmer–Lemeshow test. The validity of this nomogram model was checked on an independent external validation cohort considering discrimination and calibration. Decision-curve analysis was conducted in the validation cohort to determine the net benefit of the decision for mortality with the nomogram at different threshold probabilities. With an alpha level of 0.05, all tests were conducted two-sided.

Statistical analysis was processed by software programmes SAS (SAS v9.4; SAS Institute, NC, USA), R v3.3.3 (R Foundation for Statistical Computing, Vienna, Austria) utilizing R Studio v1.0.136 (R Studio Inc., Boston, MA, USA) and Matplotlib v3.3.4 (The Matplotlib development team) using Python 3.9 (Python Software Foundation).

## Results

### Cohort Description

#### Development Cohort

None of the 280 patients employed in the first stage were excluded because of incomplete data. As a result, a total of 280 patients, of which 42 (15.0%) died, were included in the development cohort (Table 1).

**Table 1:** Clinical and demographic data for development and validation cohort.

	**Development cohort**				**Validation cohort**			
**Characteristics**	**All**	**Non-survivors**	**Survivors**	***P-*value**	**All**	**Non-survivors**	**Survivors**	***P-*value**
	**(*n* = 280)**	**(*n* = 42)**	**(*n* = 238)**		**(*n* = 237)**	**(*n* = 43)**	**(*n* = 194)**	
Age, y	55.0 (43.8–63.0)	52.5 (24.5–66.8)	55.0 (44.0–63.0)	0.201	55.0 (44.0–63.0)	56.0 (47.0–65.0)	55.0 (43.2–62.0)	0.742
Male, *n* (%)	164 (58.6)	33 (78.6)	131 (55.0)	0.807	124 (52.3)	22 (51.2)	102 (52.6)	0.999
Hypertension, *n* (%)	9 (3.2)	1 (2.4)	9 (3.8)	0.616	13 (5.5)	1 (2.3)	12 (6.2)	0.525
DM, *n* (%)	10 (3.6)	2 (4.8)	10 (4.2)	0.367	13 (5.5)	2 (4.7)	11 (5.7)	0.917
Neurological pathogenesis, *n* (%)
Vascular	68 (24.3)	24 (57.1)	44 (18.5)	0.000	67 (28.3)	24 (55.8)	43 (22.2)	<0.001
Tumorous	157 (56.1)	3 (7.1)	154 (64.7)	0.604	99 (41.8)	5 (11.6)	94 (48.5)	0.625
Infectious	15 (5.4)	4 (9.5)	11 (4.6)	0.353	27 (11.4)	2 (4.7)	25 (12.9)	0.203
Traumatic	15 (5.4)	1 (2.4)	14 (5.9)	0.577	11 (4.6)	1 (2.3)	10 (5.2)	0.691
Metabolic	25 (8.9)	10 (23.8)	15 (6.3)	0.501	31 (13.1)	10 (23.3)	21 (10.8)	0.053
Toxic	2 (0.7)	1 (2.4)	1 (0.4)	0.765	2 (0.8)	1 (2.3)	1 (0.5)	0.801
COP, g/L	20.2 (3.1)	17.2 (3.1)	20.7 (2.8)	0.000	20.1 (3.0)	16.7(2.5)	20.8 (2.6)	<0.001
TP, g/L	58.2 (9.8)	52.1 (9.8)	59.3 (9.4)	0.000	57.2 (9.6)	48.2 (8.1)	59.2 (8.7)	<0.001
ALB, g/L	30.6 (26.6–34.9)	27.6 (22.5–30.3)	31.2 (26.9–35.2)	0.000	29.5 (25.9–34.6)	23.8 (21.9–29.2)	31.3 (27.2–35.6)	<0.001
Crystal osmotic pressure, g/L	302.8 (296.3–313.5)	304.0 (301.6–312.2)	302.2 (295.5–313.6)	0.214	308.4 (299.0–329.4)	313.8 (302.8–333.8)	308.1 (298.5–328.6)	0.864
FIB, g/L	3.4 (2.6–4.3)	4.2 (2.9–5.2)	3.3 (2.6–4.1)	0.170	3.3 (2.5–4.1)	3.1 (1.9–4.3)	3.3 (2.7–4.1)	0.277
Na, mmol/L	140.1 (137.7–143.9)	140.0 (138.7–141.8)	140.1 (137.5–144.3)	0.941	139.9 (137.5–143.9)	140.1 (138.1–143.9)	139.9 (137.4–143.9)	0.795
GLU, mmol/L	7.6 (6.3–9.9)	7.6 (6.4–9.3)	7.6 (6.3–10.0)	0.574	7.5 (6.2–9.6)	7.4 (5.6–8.4)	7.6 (6.2–10.1)	0.523
CL, mmol/L	105.8 (102.8–110.1)	104.5 (103.4–107.2)	106.2 (102.6–110.6)	0.141	106.0 (102.5–110.9)	105.3 (102.2–109.6)	106.2 (102.5–110.9)	0.277
K, mmol/L	3.7 (3.4–4.0)	3.8 (3.6–4.1)	3.7 (3.4–4.0)	0.313	3.6 (3.4–4.0)	3.7 (3.5–4.0)	3.6 (3.4–4.0)	0.856
Serum Cr, μmol/L	71.0 (56.3–93.2)	74.6 (64.0–133.0)	71.0 (55.6–88.9)	0.425	71.9 (57.0–95.0)	74.0 (59.0–87.6)	72.0 (57.0–97.0)	0.555
Bun, mg/dl	5.7 (3.8–9.5)	8.4 (4.0–11.2)	5.4 (3.7–8.7)	0.222	6.4 (3.8–9.7)	6.4 (3.9–9.4)	6.2 (3.8–9.7)	0.501
Ca, mmol/L	2.1 (2.0–2.2)	2.1 (2.0–2.2)	2.1 (2.0–2.2)	0.964	2.1 (2.0–2.2)	2.1 (2.0–2.2)	2.1 (2.0–2.2)	0.679
APACHE II score	11.0 (8.0–18.0)	20.5 (13.0–23.0)	9.5 (8.0–16.0)	0.000	11.0 (8.0–19.0)	21.0 (15.0–23.0)	10.0 (8.0–16.0)	<0.001
GCS score	12.0 (11.0–15.0)	10.0 (7.0–14.0)	14.0 (13.0–15.0)	0.000	13.0 (11.0–15.0)	8.0 (5.0–11.0)	14.0 (13.0–15.0)	<0.001
Duration of ventilation, h	4.0 (3.0–108.5)	196.0 (144.0–264.0)	4.0 (3.0–61.0)	0.000	4.0 (3.0–96.0)	144.0 (36.0–228.0)	4.0 (3.0–46.0)	<0.001
Length of stay, d	1.0 (1.0–5.2)	10.0 (7.0–14.0)	1.0 (1.0–4.0)	0.000	1.0 (1.0–5.0)	6.0 (1.5–11.0)	1.0 (1.0–4.0)	<0.001

*Data presented as median (IQR) or n (%). Length of stay was defined as the length of stay in the neurointensive care unit (NICU), emergency intensive care unit (EICU), or emergency department. ALB, albumin; APACHE II, Acute Physiology and Chronic Health Evaluation Score II; BUN, blood urea nitrogen; Ca, calcium; Cl, chlorine; COP, colloid osmotic pressure; Cr, creatinine; FIB, fibrinogen; DM, Diabetes mellitus; GCS, Glasgow Coma Scale; ICU, intensive care unit; IQR, interquartile range; K, potassium; Na, sodium; TP, total protein*.

#### Validation Cohort

Of the 237 patients who were eligible in the second stage, none were excluded because of incomplete data. Thus, a total of 237 patients, of which 43 (18.1%) died, were included in the validation cohort (Table 1).

### Development of the Nomogram Model

**Figure 1:**
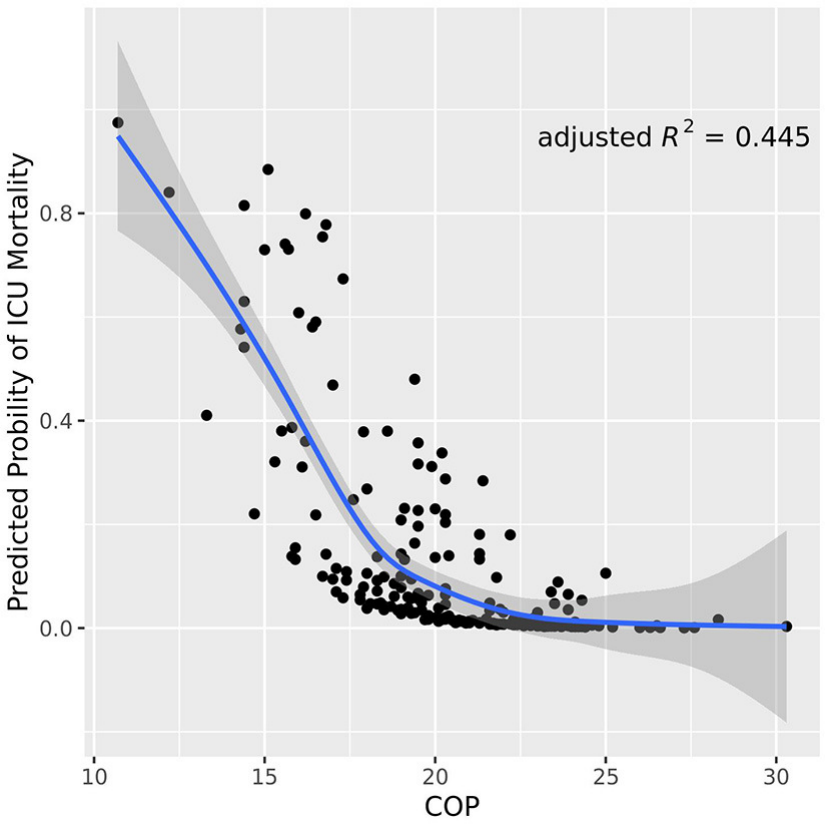
Scatter plot of COP values correlated to neurological ICU mortality. The blue line indicates the trend line with a gray shadow representing the 95% confidence interval. The plot visualizes that the mortality risks descends as the COP values ascend in neurological ICU patients. COP, colloid osmotic pressure.

As shown in Table 1, variables presenting significance including COP, neurological pathogenesis of vascular, sTP, sALB and scoring systems (APACHE II score and GCS score) were selected to the multivariate logistic regression. COP, neurological pathogenesis and APACHE II score were recognized as independent predictors in the multivariate logistic regression analysis (Table 2). Patients with neurological pathogenesis of vascular (OR, 7.062; 95% CI, 2.357–21.157; *P* <0.001) or higher APACHE II score (OR, 1.095; 95% CI, 1.022–1.174; *P* = 0.01) had higher probabilities of death. By contrast, the higher the COP (OR, 0.598; 95% CI, 0.482–0.742; *P* < 0.001), the less likely the death was to be achieved (Table 2). As also presented in Figure 1, the lower the COP value, the higher the risk of death will be. A linear correlation was visualized between NICU mortality and COP values with an adjusted R square of 0.445. Obviously, with the increase of COP, mortality decreased to a relatively lower valley, with a trend that when the COP values climbed over 20 mmHg, the mortality seemed stay no more higher.

**Table 2:** Multivariate logistic regression analysis of predictors for the ICU death.

**Influencing**	**OR (95%CI)**	***P-*value**
**factors**		
COP	0.598 (0.482–0.742)	<0.001
APACHE II score	1.095 (1.022–1.174)	0.01
Type of neurological disease		0.002
Tumorous/infectious/traumatic/ metabolic/poisonous	1.000 (referent)	
Vascular	7.062 (2.357–21.157)	<0.001

*APACHE II, Acute Physiology and Chronic Health Evaluation Score II; COP, colloid osmotic pressure; ICU, intensive care unit; OR, Odds ratio; CI, Confidence interval*.

**Figure 2:**
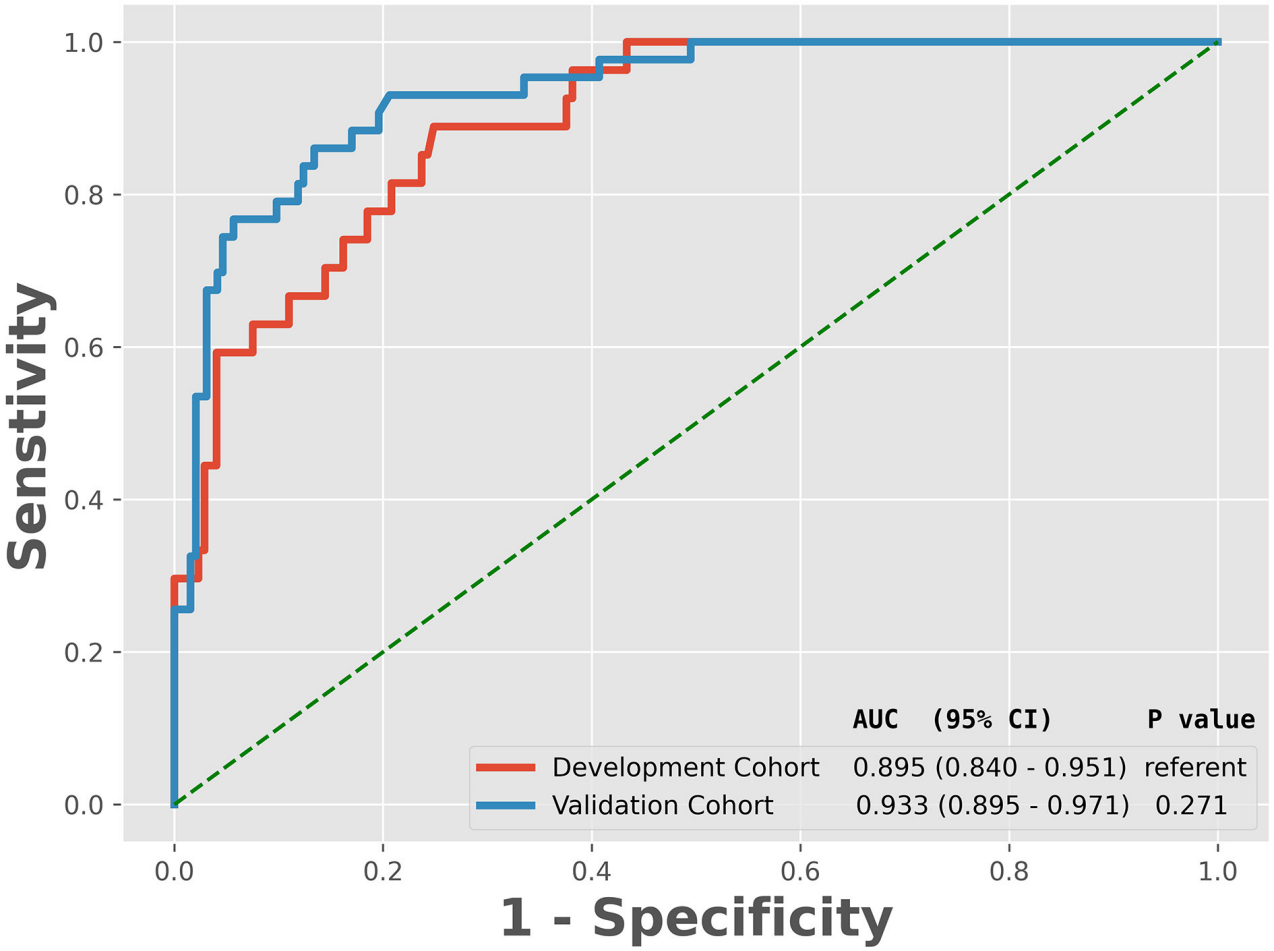
Receiver operating characteristic curve analyses of prediction for the mortality in the development and validation cohort. AUC, the area under the receiver operating characteristic curve; CI, confidence interval.

**Figure 3:**
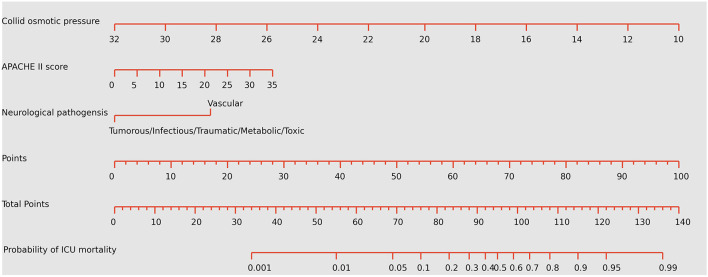
Nomogram predicting the probabilities of mortality for neurological critically ill patients. To obtain the nomogram-predicted probability, locate patient values on each axis. Draw a vertical line to the point axis to determine how many points are attributed for each variable value. Sum the points for all variables. Locate the sum on the total point line to assess the individual probability of mortality in the neurointensive care unit. APACHE II, Acute Physiology and Chronic Health Evaluation II. The unit of colloid osmotic pressure is mmHg.

**Figure 4:**
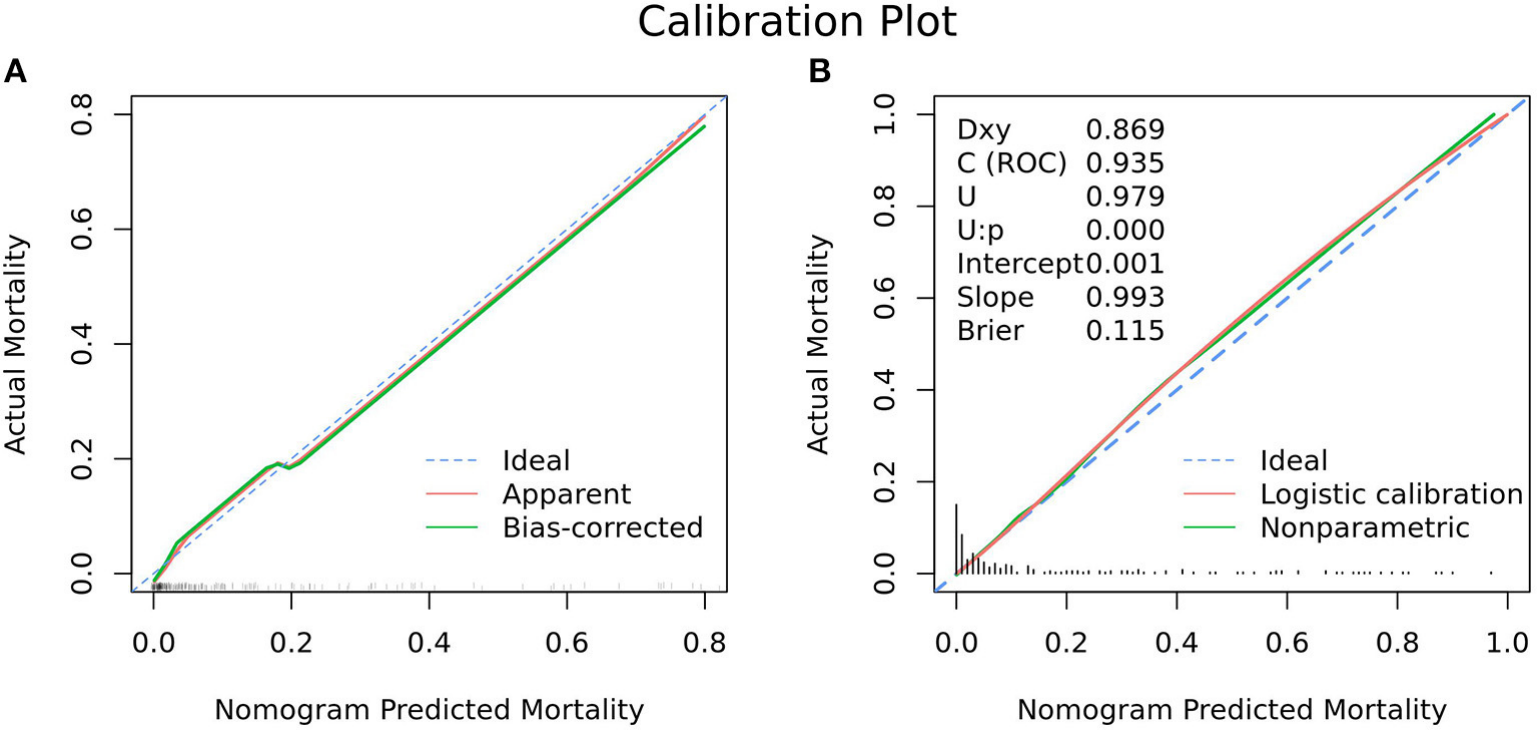
Calibration plot for nomogram in the **(A)** development cohort and **(B)** validation cohort. **(A)** The 45° dashed line (“Ideal”) represents ideal predictions, the plot illustrates the accuracy of the best-fit model (“Apparent”) and the bootstrap model (“Bias-corrected”) for predicting ICU mortality. The ticks across the x-axis represent the frequency distribution of the predicted probabilities. **(B)** The blue dashed line denotes perfect calibration. A smoothing curve (green solid line) and the calibration curve (red solid line) are also overlaid. The distribution of calculated predicted probabilities is overlaid along the horizontal axis. A subset of various statistics useful for validating the model are also shown. Dxy: Somers' Dxy rank correlation between *p* (predicted possibilities) and y (actual outcome = 0 or 1). C(ROC): the ROC area. U: Unreliability index, for testing unreliability. Brier: Brier score, average squared difference in *p* (predicted possibilities) and y (actual outcome = 0 or 1).

The ROC analysis of predictors of death in the development cohort is demonstrated in Figure 2. The prediction model that incorporated the identified predictors was completed and presented as the nomogram (Figure 3). With high accuracy, high sensitivity, high NPV, low specificity and low PPV in the development cohort, this development model is considered highly effective in identifying patients with a lower risk of mortality (Table 3). Calibration plots for the nomogram in the development cohort were generated (Figure 4A).

**Table 3:** Detective characteristics of the development and validation cohort.

**Cohort**	**AUC (95%CI)**	**Accuracy (95%CI)**	**Sensitivity (95%CI)**	**Specificity (95%CI)**	**NPV (95%CI, %)**	**PPV (95%CI, %)**
Development	0.895 (0.840–0.951)	0.724 (0.655–0.786)	0.962 (0.819–0.993)	0.679 (0.614–0.749)	99.3 (95.2–99.8)	32.5 (22.4–43.7)
Validation	0.934 (0.892–0.976)	0.753 (0.678–0.812)	0.941 (0.812–0.994)	0.715 (0.625–0.782)	98.4 (94.4–99.2)	44.7 (32.5–55.8)

*AUC, area under the receiver operating characteristic curve; CI, confidence interval; NPV, negative predictive value; PPV, positive predictive value*.

### Validation of the Nomogram Model

As revealed by Table 1, variables absorbed in the multivariate logistic regression due to significance in the validation cohort were the same as those in the development cohort. As for the clinical outcomes, patients who died had a longer duration of ventilation and longer length of stay than survivors in both the development cohort and validation cohort (Table 1). The ROC analysis of the predictors of mortality in the validation cohort is demonstrated in Figure 2. With AUCs (95% CI) of 0.895 (0.840–0.951) and 0.934 (0.892–0.976), no significant difference was found between the development and validation cohorts by using the DeLong test with a *P*-value of 0.274 (Figure 2). Similar to the development cohort, this validation model highly effectively identified patients with a lower risk of mortality, due to its corresponding high accuracy, high sensitivity, high NPV, low specificity and low PPV (Table 3). With the AUC of 0.935, the ability to predict mortality in the validation cohort was ideal (Figure 4B). Besides, the model in the validation cohort presented good consistency, as Figure 4B displayed.

### Clinical Use

**Figure 5:**
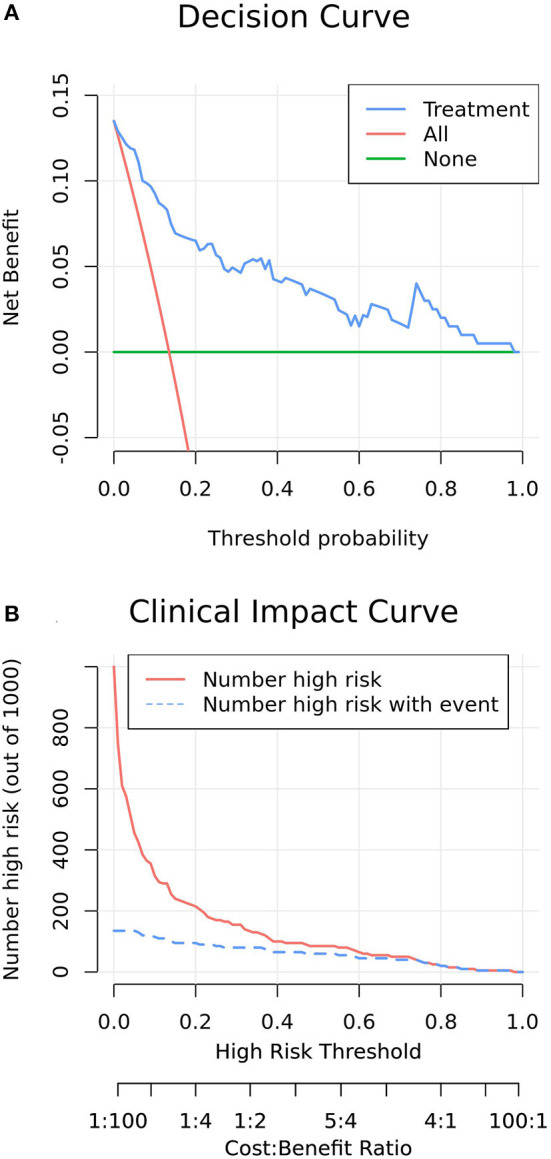
**(A)** Decision curve analysis of increasing COP in patients with the model nomogram. The y-axis measures the net benefit. The green line represents the model nomogram. The blue long-dashed line represents the assumption that all patients undertake post-pyloric tube placement. Thin red dashed line represents the assumption that no post-pyloric patient undertakes tube placement. The net benefit was calculated by subtracting the proportion of all patients who are false positive from the proportion who are true positive, weighting by the relative cost of forgoing administration compared with the negative consequences of an unnecessary administration. Threshold probability is the probability of survival from which an intensivist considers that he decides an intervention measure to increase the COP. The decision curve showed that if the threshold probability of a ICU survival is 5% or above, which explicitly covers the range of clinically reasonable threshold probabilities (probability of success >50%), using the nomogram in the current study to predict ICU survival adds more benefit than the administer-all scheme or the administer-none scheme. For example, if the personal threshold probability of a ICU survival is 50% (i.e., the intensivist would opt for administration if the patient's probability was 50%), then the net benefit is 0.045 when using the nomogram to make the decision of whether to start the administration, with added benefit than the administer-all scheme or the administer-none scheme. **(B)** Clinical impact curve of model nomogram. The red curve (number of high-risk individuals) indicates the number of people who are Classified as positive (high risk) by the model at each threshold probability; the blue curve (number of high-risk individuals with outcome) is the number of true positives at each threshold probability. Clinical impact curve visually indicated that nomogram conferred high clinical net benefit and confirmed the clinical value of the nomogram.

Figure 5A presents the decision-curve analysis of this nomogram. Threshold probability is defined as the probability of mortality from which a physician is viewed in the NICU, EICU or emergency department. As shown in the decision curve, regardless of the threshold probability of mortality, it is more efficient to use this predictive nomogram to predict mortality than other alternative strategies. The bootstrap method was used to simulate 1,000 patients with high-risk status. When the event incidence rate was >60%, the event occurrence probability was utterly consistent with the model, suggesting that the model had good prediction performance (Figure 5B). According to the economic benefit ratio, the earlier the intervention was given to patients with high-risk status, the better the economic benefit ratio (Figure 5B).

## Discussion

To date, there have been few prospective studies regarding mortality in patients with critical neurological status across the spectrum. Therefore, we performed a two-stage, duocentric prospective study to assess mortality in critically ill neurological patients. Our results show a mortality rate of up to 15.0% in the development cohort and 18.1% in the validation cohort. Thus, our study provides additional evidence for mortality in this population group. Likewise, this study confirmed that COP, APACHE II score and neurological pathogenesis are independent predictors and established a practical and straightforward nomogram for predicting individual mortality in critically ill neurological patients. Lower concentration of COP, higher APACHE II score and neurological pathogenesis of cerebrovascular diseases facilitated the death of patients with critical neurological disorders. To the best of our knowledge, this study is the first attempt to develop a predictive nomogram for mortality of patients with critical neurological status based on clinical data.

Brain swelling can force brain tissues to move into abnormal anatomical spaces under pressure, causing damage to these brain areas and even brain hernia. However, with the development of intensive care techniques, clinicians can titrate therapy based on objective information collected from monitors. Therefore, for many clinical applications, ICP monitoring is required, but non-invasive monitoring is necessary under certain circumstances, such as in conscious patients (i.e., ophthalmology, neurology, aerospace medicine cases, etc.). Unfortunately, in clinical practice, many non-invasive monitoring techniques lack accuracy. A study also showed that decreased plasma COP and serum protein levels can be used to indicate the severity of injury in patients with trauma (14). Therefore, COP can be used in place of ICP for intracranial pressure monitoring in specific cases, which is expected to play a role in the mortality prediction of patients with critical neurological status. On this basis, this study was conducted and found that COP is a good predictor of mortality in patients with critical neurological disorders. Notably, COP has more comprehensive indications, fewer contraindications than ICP, making it safer and more extensive clinical application. For example, COP can be used as a screening tool in emergency departments for patients who are suspected of needing invasive monitoring of ICP.

The APACHE II score can be a very promising prognostic predictor that can assess the patient's overall health status and correlate with disease severity (22, 23). Furthermore, the GCS (24) and APACHE II scores were reported as predictors of prognosis for many medical conditions such as traumatic brain injury (25). Consistently, our study revealed that the APACHE II score occupied a large proportion in this predictive nomogram. Previous studies had also demonstrated that the GCS score could be utilized in predicting adverse outcomes since it is valuable in identifying consciousness and neurological dysfunction (26, 27). Thus, the GCS score is conceivable to use as a potential predictor. However, the multivariate model eliminated the GCS score, although it was significant in the univariate analysis. Furthermore, the GCS and APACHE II scores share most of the same scoring criteria, resulting in the collinearity between the two scoring systems. Hence, our study has recognized that the APACHE II score is an essential predictor of mortality probability.

This study presents that patients with cerebrovascular diseases are vulnerable to a higher mortality probability than those exposed to tumorous, infectious, traumatic, metabolic and fatal neurological diseases. Cerebrovascular diseases, which have high disability and mortality rates, are becoming increasingly common worldwide. Cerebrovascular disease is a leading cause of death because of its acute progression, quick deterioration and severe complications, such as cerebral oedema (28). With high morbidity, disability rates and death rates, cerebrovascular disease hurts the quality of life and public health (29).

Unlike a previous study explaining that age, diagnosis, GCS, pupillary status, sALB and serum sodium are independent predictors of survival in patients with critical neurological status (30), our study has the following merits. Firstly, factors included in our model and complex clinical algorithms are substantially reduced, making our model convenient and straightforward to speculate the mortality rate; thus, the clinical model becomes more widely used in clinical practice. Secondly, our model's APACHE II scoring system can more comprehensively reflect patients' overall situation and enhance the prediction performance. Moreover, the APACHE II scoring system has been routinely used to evaluate the overall condition and predict the prognosis of patients in clinical application. Meanwhile, with the development of artificial intelligence, real-time automatic data acquisition and updates of the APACHE II scoring system have been realized. Therefore, the existing clinical scoring system no longer has the disadvantage of the complex algorithm, so it cannot be promoted and used. Thirdly, prediction by COP, which was included in our model, was better than the sALB prediction. Although sALB, sTP and COP demonstrated significant differences in the univariate analysis, COP showed better performance in the multivariate analysis. Serum ALB and sTP were excluded in the multivariate analysis probably because there was collinearity with COP, and the sALB and sTP might have been affected by more confounding factors. Fourthly, since many critically ill neurological patients were included in our study, the etiologies were complex. Thus, the external scalability was increased. Fifthly, the etiology, biological markers and clinical factors were included in our predictive model, which could comprehensively and accurately assess the condition and decide the clinical diagnosis and treatment. Moreover, a study presented that the combination of clinical markers and biomarkers is an effective method for the accurate prediction of prognosis (31). Finally, the capillary state assessed in previous studies is affected by various factors, which will influence the accuracy of prediction performance.

Furthermore, owing to its visualizing, intuitive and appreciable specialties, the nomogram was gradually accepted and consolidated to facilitate decision-making in the clinic, especially for individualized therapy (32, 33). The guideline encourages physicians to conduct clinical research involving standardized clinical management across centers, independent monitoring of patient management and data quality, minimal data collection, determination of relevant outcome measures and adequate follow-up time and clinically relevant magnitude of influence (34). The influence of relevant clinical factors can be determined by using the nomogram model, which can reduce data collection, improve the accuracy of diagnosis and treatment, estimate patients' prognosis and promote standardized clinical management.

Bedside computerized and graphical decision instruments comprising decision trees, nomograms and machine learning have acquired wide acceptance to advance monitoring, risk classification, diagnosis, therapy and prognosis in critical care (35–38). However, decision trees tend to be unstable and relatively inaccurate, especially for data that contain variables of different levels of categories, even if they are easy to understand. Nomograms thrive in various environments because they allow fast and accurate calculations without the need for calculators. Predictive nomograms are derived from clinically available factors and provide a viewable, easy-to-use scale for predicting probability (36). Nomograms produce reliable results very rapidly, requiring only one or more lines to be drawn without substituting numbers into the equation. In this study, the predictive factors determined by logistic regression were incorporated into the prediction model, and ultimately, the clinically validated practicable nomogram was depicted based on the model.

The primary purpose of using predictive nomograms was to identify individual mortality during hospitalization. However, the predictive performance of identification and calibration does not fully stand for the clinical validity on a particular level of identification or degree of calibration. Therefore, to verify the clinical utility of this nomogram, we evaluated whether the use of nomograms to aid decision-making improved the predictive power of mortality. Accordingly, we used decision-curve analysis to validate nomograms in a prospective cohort. This novel approach generated perception into clinical prognoses grounded on threshold probability, from which a net benefit can be derived. Furthermore, the decision curve presented that nomograms developed in the present study to predict mortality enable provision of more enhanced services than any other alternative strategy, regardless of the threshold probability of death. Thus, the nomogram will not only greatly facilitate informed decision-making by critical care physicians but also provide survival or mortality benefits for the patients. The importance of this predictive model in research and resource management applications cannot be underestimated. Whether guided by nomograms or not, further studies of cost-effectiveness analysis concerning strategies to improve predictive power should be performed before widespread clinical application.

This study has the following benefits and strength. First, a two-stage design that demonstrated that the same disease could be verified in different scenarios, times and locations was used to display consistent results in the development and validation cohorts, suggesting that our study's internal validation and external scalability were promising. Second, our method has several potential clinical applications. For example, COP measurements may conveniently assess ICP or systemic conditions, especially when the device has malfunctioned or unavailable for plug-in invasive monitoring. For instance, it can be used to monitor patients at risk for intracranial hypertension during monitoring. Third, with further development of technology, COP can be realized at the bedside and in continuous real-time monitoring. Thus, the ability of the COP to predict mortality in the NICU may be underrated. Moreover, both experimental evidence and theoretical foundation for the advance of clinical therapy were provided in this study, and its prospect and potential clinical application are worthy of further research. However, the limitation also occurred in this study. Although the number of subjects obtained exceeded the calculated sample size, the number of patients used to establish the predictive model was relatively small. Furthermore, since we used the same device for COP measurement, we only conducted this experiment in two centers. Therefore, the prediction accuracy of this nomogram may be underestimated or overestimated in this study.

## Conclusion

By incorporating COP, neurological pathogenesis and APACHE II score in the model, this useful nomogram could be accessibly utilized to predict mortality in critically ill neurological patients. With the high mortality rate in patients with critical neurological impairment, this predictive nomogram can play an essential role in determining the best treatment and discharge protocol by identifying relevant factors of mortality.

## Data Availability Statement

The raw data supporting the conclusions of this article will be made available by the authors, without undue reservation.

## Ethics Statement

The Ethics Committee of the Guangdong Provincial People's Hospital and Longgang District Central Hospital approved the protocol. Written informed consent was obtained from each patient or from appropriate surrogates for patients unable to consent.

## Author Contributions

BL, LHu, YH, and CC contributed to the conception, design of the research, interpretation of the data, and critically revised the manuscript. BL, HF, JD, JX, HZ, LHe, and YL performed the study and collected data. BL, YH, DS, and LHu analyzed the data. All authors contributed to the acquisition and analysis of the data, drafted the manuscript, agree to be fully accountable for ensuring the integrity and accuracy of the work, and read and approved the final manuscript.

## Funding

CC is currently receiving a grant (#number of MaoRenCaiBan[2020]24) from the Office of Talent Work Leading Group in Maoming, a grant (#201803010058) from the Guangzhou Science and Technology Program, and a grant (#DFJH2020028) under the Major Program of Summit Project, Guangdong Province High-level Hospital Construction Project of Guangdong Provincial People's Hospital, Guangdong Academy of Medical Sciences. LHu is currently receiving a grant (#2020YJ01) from the Emergent Science and Technology Project for Prevention and Treatment of Novel Coronavirus Pneumonia and a grant (#zx2020017) from the High-level Hospital Construction Research Project of Maoming People's Hospital. The study was supported by the High-level Hospital Construction Research Project of Maoming People's Hospital.

## Conflict of Interest

The authors declare that the research was conducted in the absence of any commercial or financial relationships that could be construed as a potential conflict of interest.

## Publisher's Note

All claims expressed in this article are solely those of the authors and do not necessarily represent those of their affiliated organizations, or those of the publisher, the editors and the reviewers. Any product that may be evaluated in this article, or claim that may be made by its manufacturer, is not guaranteed or endorsed by the publisher.

## Abbreviations

ALB: albumin
APACHE II: Acute Physiology and Chronic Health Evaluation II
AUC: area under the receiver operating characteristic curve
BUN: blood urea nitrogen
Ca: calcium
CI: confidence interval
Cl: chlorine
COP: colloid osmotic pressure
EICU: emergency intensive care unit
FIB: fibrinogen
GCS: Glasgow Coma Scale
Glu: glucose
Hb: hemoglobin
ICP: intracranial pressure
K: potassium
Na: sodium
NPV: negative predictive values
OR: odds ratios
P-POSSUM: Portsmouth predictor equation-Physiology and Operative Severity Score for enUmeration of Mortality and Morbidity
PPV: positive predictive values
ROC: receiver operating characteristic
sALB: serum albumin
SAPS II: Simplified Acute Physiology Score II
sCr: serum creatinine
sTP: Serum total protein
TP: total protein.

## References

[1] Kramer AH Zygun DA. Declining mortality in neurocritical care patients: a cohort study in Southern Alberta over eleven years. Can J Anaesth. (2013) 60:966–75. 10.1007/s12630-013-0001-0 23877315

[2] Farahvar A Gerber LM Chiu YL Härtl R Froelich M Carney N. Response to intracranial hypertension treatment as a predictor of death in patients with severe traumatic brain injury. J Neurosurg. (2011) 114:1471–8. 10.3171/2010.11.JNS101116 21214327

[3] Gu JJ Huang HP Huang YJ Sun HT Xu HW. Hypertonic saline or mannitol for treating elevated intracranial pressure in traumatic brain injury: a meta-analysis of randomized controlled trials. Neurosurg Rev. (2019) 42:499–509. 10.1007/s10143-018-0991-8 29905883

[4] Deng YY Shen FC Xie D Han QP Fang M Chen CB. Progress in drug treatment of cerebral edema. Mini Rev Med Chem. (2016) 16:917–25. 10.2174/1389557516666160304151233 26948324

[5] Yuan Q Wu X Cheng HW Yang CH Wang YH Wang ES. Is intracranial pressure monitoring of patients with diffuse traumatic brain injury valuable? An observational multicenter study. Neurosurgery. (2016) 78:361–8. 10.1227/NEU.0000000000001050 26891376

[6] Zou ZH Zhang SZ Wang KQ Wu QF Yin M Wang JJ. Clinical importance of non-invasive intracranial pressure monitoring in early prediction of hematoma enlargement following hypertensive intracerebral haemorrhage. Acta Medica Mediterr. (2013) 29:295–9. Available online at: https://www.actamedicamediterranea.com/archive/2013/medica-2/clinical-importance-of-non-invasive-intracranial-pressure-monitoring-in-early-prediction-of-hematoma-enlargement-following-hypertensive-intracerebral-haemorrhage/document

[7] Hemphill JC III Greenberg SM Anderson CS Becker K Bendok BR Cushman M. Guidelines for the management of spontaneous intracerebral hemorrhage: a guideline for healthcare professionals from the american heart association/american stroke association. Stroke. (2015) 46:2032–60. 10.1161/STR.0000000000000069 26022637

[8] Potapov AA Krylov VV Gavrilov AG Kravchuk AD Likhterman LB Petrikov SS. Guidelines for the diagnosis and treatment of severe traumatic brain injury. Part 2. Intensive care and neuromonitoring. Zh Vop Neirokhir Im N N Burdenko. (2016) 80:98–106. 10.17116/neiro201680198-106 27029336

[9] Dawes AJ Sacks GD Cryer HG Gruen JP Preston C Gorospe D. Intracranial pressure monitoring and inpatient mortality in severe traumatic brain injury: a propensity score-matched analysis. J Trauma Acute Care Surg. (2015) 78:492–501. 10.1097/TA.0000000000000559 25710418

[10] Ramesh VJ Umamaheswara Rao GS Kandavel T Kumaraswamy SD Iyyamanda UB Chandramouli BA. Predictive model for survival among neurosurgical intensive care patients. J Neurosurg Anesthesiol. (2011) 23:183–7. 10.1097/ANA.0b013e31821cb9ec 21593685

[11] Kish JL McGuirk SM Friedrichs KR Peek SF. Defining colloid osmotic pressure and the relationship between blood proteins and colloid osmotic pressure in dairy cows and calves. J Vet Emerg Crit Care. (2016) 26:675–81. 10.1111/vec.12517 27599220

[12] Ookawara S Sato H Takeda H Tabei K. Method for approximating colloid osmotic pressure in long-term hemodialysis patients. Ther Apheresis Dial. (2014) 18:202–7. 10.1111/1744-9987.12070 24720412

[13] Martin GS. Fluid balance and colloid osmotic pressure in acute respiratory failure: emerging clinical evidence. Crit Care. (2000) 4(Suppl. 2):S21–5. 10.1186/cc966 11255595 PMC3226171

[14] Rahbar E Baer LA Cotton BA Holcomb JB Wade CE. Plasma colloid osmotic pressure is an early indicator of injury and hemorrhagic shock. Shock. (2014) 41:181–7. 10.1097/SHK.0000000000000101 24280690

[15] Cho DY Wang YC. Comparison of the APACHE III, APACHE II and Glasgow Coma Scale in acute head injury for prediction of mortality and functional outcome. Intensive Care Med. (1997) 23:77–84. 10.1007/s001340050294 9037644

[16] Schuiling WJ de Weerd AW Dennesen PJ Algra A Rinkel GJ. The simplified acute physiology score to predict outcome in patients with subarachnoid hemorrhage. Neurosurgery. (2005) 57:230–36. 10.1227/01.NEU.0000166536.42876.9C 16094150

[17] Deng Y Yuan J Chi R Ye H Zhou D Wang S. The incidence, risk factors and outcomes of postoperative acute kidney injury in neurosurgical critically ill patients. Sci Rep. (2017) 7:4245. 10.1038/s41598-017-04627-3 28652590 PMC5484679

[18] Lv B Hu L Chen L Hu B Zhang Y Ye H. Blind bedside postpyloric placement of spiral tube as rescue therapy in critically ill patients: a prospective, tricentric, observational study. Crit Care. (2017) 21:248. 10.1186/s13054-017-1839-2 28950897 PMC5615440

[19] Peduzzi P Concato J Feinstein AR Holford TR. Importance of events per independent variable in proportional hazards regression analysis. II. Accuracy and precision of regression estimates. J Clin Epidemiol. (1995) 48:1503–10. 10.1016/0895-4356(95)00048-8 8543964

[20] Wu X Li Y Jin X Liu Y Zang W. Post-operative change in colloid osmotic pressure and its clinical significance after heart surgery in adults. Biomed Res. (2017) 28:4. Available online at: https://www.alliedacademies.org/articles/postoperative-change-in-colloid-osmotic-pressure-and-its-clinical-significance-after-heart-surgery-in-adults.pdf

[21] Klanderman RB Bosboom JJ Korsten H Zeiler T Musson REA Veelo DP. Colloid osmotic pressure of contemporary and novel transfusion products. Vox Sanguinis. (2020) 115:664–75. 10.1111/vox.12932 32378239 PMC7754447

[22] Yoon JC Kim YJ Lee YJ Ryoo SM Sohn CH Seo DW. Serial evaluation of SOFA and APACHE II scores to predict neurologic outcomes of out-of-hospital cardiac arrest survivors with targeted temperature management. PLoS ONE. (2018) 13:e0195628. 10.1371/journal.pone.0195628 29621337 PMC5886591

[23] Wong DT Crofts SL Gomez M McGuire GP Byrick RJ. Evaluation of predictive ability of APACHE II system and hospital outcome in Canadian intensive care unit patients. Crit Care Med. (1995) 23:1177–83. 10.1097/00003246-199507000-00005 7600824

[24] Rosenfeld JV Maas AI Bragge P Morganti-Kossmann MC Manley GT Gruen RL. Early management of severe traumatic brain injury. Lancet. (2012) 380:1088–98. 10.1016/S0140-6736(12)60864-2 22998718

[25] Park SK Chun HJ Kim DW Im TH Hong HJ Yi HJ. Acute Physiology and Chronic Health Evaluation II and Simplified Acute Physiology Score II in predicting hospital mortality of neurosurgical intensive care unit patients. J Korean Med Sci. (2009) 24:420–6. 10.3346/jkms.2009.24.3.420 19543503 PMC2698186

[26] Teasdale G Jennett B. Assessment of coma and impaired consciousness. A practical scale. Lancet. (1974) 2:81–4. 10.1016/S0140-6736(74)91639-0 4136544

[27] Balestreri M Czosnyka M Chatfield DA Steiner LA Schmidt EA Smielewski P. Predictive value of Glasgow Coma Scale after brain trauma: change in trend over the past ten years. J Neurol Neurosurg Psychiatry. (2004) 75:161–2. Available online at: https://jnnp.bmj.com/content/jnnp/75/1/161.full.pdf 14707332 PMC1757441

[28] Mehta RI Ivanova S Tosun C Castellani RJ Gerzanich V Simard JM. Sulfonylurea receptor 1 expression in human cerebral infarcts. J Neuropathol Exp Neurol. (2013) 72:871–83. 10.1097/NEN.0b013e3182a32e40 23965746 PMC3771575

[29] Truelsen T Begg S. The Global Burden of Cerebrovascular Disease. Geneva: World Health Organization (2006).

[30] Habermehl C Schmitz CH Steinbrink J. Contrast enhanced high-resolution diffuse optical tomography of the human brain using ICG. Optics Express. (2011) 19:18636–44. 10.1364/OE.19.018636 21935232 PMC3482886

[31] Ma J Deng Y Lao H Ouyang X Liang S Wang Y. A nomogram incorporating functional and tubular damage biomarkers to predict the risk of acute kidney injury for septic patients. BMC Nephrol. (2021) 22:176. 10.1186/s12882-021-02388-w 33985459 PMC8120900

[32] Boer KR van Ruler O van Emmerik AA Sprangers MA de Rooij SE Vroom MB. Factors associated with posttraumatic stress symptoms in a prospective cohort of patients after abdominal sepsis: a nomogram. Intensive Care Med. (2008) 34:664–74. 10.1007/s00134-007-0941-3 18197398 PMC2271079

[33] Brown G Dodek P. Intravenous insulin nomogram improves blood glucose control in the critically ill. Crit Care Med. (2001) 29:1714–9. 10.1097/00003246-200109000-00010 11546970

[34] Carney N Totten AM O'Reilly C Ullman JS Hawryluk GW Bell MJ. Guidelines for the management of severe traumatic brain injury, fourth edition. Neurosurgery. (2017) 80:6–15. 10.1227/NEU.0000000000001432 27654000

[35] van IM de Vries L van den Born J Buter H Navis G Boerma C. Renal function is a major determinant of ICU-acquired hypernatremia: a balance study on sodium handling. J Transl Intern Med. (2020) 8:165–76. 10.2478/jtim-2020-0026 33062593 PMC7534501

[36] Hu L Nie Z Zhang Y Zhang Y Ye H Chi R. Development and validation of a nomogram for predicting self-propelled postpyloric placement of spiral nasoenteric tube in the critically ill: mixed retrospective and prospective cohort study. Clin Nutr. (2019) 38:2799–805. 10.1016/j.clnu.2018.12.008 30579668

[37] Chen W Sun C Wei R Zhang Y Ye H Chi R. Establishing decision trees for predicting successful postpyloric nasoenteric tube placement in critically ill patients. JPEN J Parenter Enteral Nutr. (2018) 42:132–8. 10.1177/0148607116667282 29505136

[38] Martins CB De Bels D Honore PM Redant S. Early prediction of acute kidney injury by machine learning: should we add the urine output criterion to improve this new tool? J Transl Intern Med. (2020) 8:201–2. 10.2478/jtim-2020-0031 33511045 PMC7805289

